# Supplementary material for: Dietary Supplementation of Novel Aflatoxin Oxidase CotA Alleviates Aflatoxin B1-Induced Oxidative Stress, Lipid Metabolism Disorder, and Apoptosis in the Liver of Japanese Quails
Source: Animals (Basel). 2025 May 26;15(11):1555. doi: 10.3390/ani15111555 (PMC12153666; doi:10.3390/ani15111555)
Supplement: Supplementary file 1 [file animals-15-01555-s001.zip › animals-3625125-supplementary.pdf]

Table S1. Ingredients and nutrient contents of the basal diet.

Ingredient	%
Corn	51.80
Soybean meal	36.70
Corn gluten meal	5.21
Soybean oil	2.90
Limestone	0.70
Di-calcium phosphate	1.65
NaCl	0.30
Premix [1]	0.30
L-Lysine	0.13
DL-Methionine	0.11
Choline chloride	0.20
Total	100
Calculated analysis	
Metabolizable energy (MJ/kg)	12.54
Crude protein	24.00
Calcium	0.80
Available phosphorus	0.45
Lysine	1.30
Methionine + cystine	0.92

[1] Provides per kg of diet: vitamin A, 12,000 IU; vitamin D3, 5,000 IU; vitamin E, 130.0 mg; vitamin K3, 3.605 mg; vitamin B1, 3.0 mg; vitamin B2, 8.0 mg; vitamin B6, 4.950 mg; vitamin B12, 17.0 mg; niacin, 60.0 mg; D-biotin, 200.0 mg; calcium D-pantothenate, 18.333 mg; folic acid, 2.083 mg; manganese, 100.0 mg; iron, 80.0 mg; zinc, 80.0 mg; copper, 8.0 mg; iodine, 2.0 mg; cobalt, 500.0 mg; and selenium, 150.0 mg.

Table S2. Primers used in real-time quantitative PCR.

Gene		Primer Sequence（5'–3'）	（bp）	Accession number
Nrf2	F:	TGGAGCCAGGCACTCAAAAA	115	XM_015868399.2
	R：	CGGCCCGAAGGCTTATCTTT		
HO-1	F:	CACGGCATCCCTGTACTTCA	119	XM_015863488.2
	R：	CCAGGGCATCTTTCCGATGT		
NQO1	F:	CCACCATGTATGACCAGGGG	82	XM_015874307.1
	R：	CTGTACATGGAGCCCATCCC		
SOD1	F:	AGCACGGTGGACCAAAAGAT	135	XM_015881247.1
	R：	TACGGCCAATGATGCAGTGT		
Bcl2	F:	CCGCTACCAGAGGGACTTTG	114	XM_015854614.2
	R：	CAGTTGACCCCATCACGGAA		
Caspase 3	F:	AGAAGTCTGGCAGGGAAACC	99	XM_015861407.1
	R：	CGTTTCATCTGGTCCGCTGT		
Caspase 9	F:	GTGCCCGAGTTTGAGAGGAA	83	XM_015882460.2
	R：	CCACCTCGAATCCTTGGTCC		
Bax	F:	CTCAAGGCCCTGTGCACTAA	128	NM_017059.2
	R：	TAGGAAAGGAGGCCATCCCA		
β-actin	F:	ATGATATTGCTGCGCTCGTTG	127	XM_015876619.1
	R：	ACCATCACACCCTGATGTCTG		
